# Topological constraints are major determinants of tRNA tertiary structure and dynamics and provide basis for tertiary folding cooperativity

**DOI:** 10.1093/nar/gku807

**Published:** 2014-09-12

**Authors:** Anthony M. Mustoe, Charles L. Brooks, Hashim M. Al-Hashimi

**Affiliations:** 1 Department of Biophysics, University of Michigan, Ann Arbor, MI 48109, USA; 2 Department of Chemistry, University of Michigan, Ann Arbor, MI 48109, USA; 3 Department of Biochemistry and Chemistry, Duke University School of Medicine, Durham, NC 27710, USA

## Abstract

Recent studies have shown that basic steric and connectivity constraints encoded at the secondary structure level are key determinants of 3D structure and dynamics in simple two-way RNA junctions. However, the role of these topological constraints in higher order RNA junctions remains poorly understood. Here, we use a specialized coarse-grained molecular dynamics model to directly probe the thermodynamic contributions of topological constraints in defining the 3D architecture and dynamics of transfer RNA (tRNA). Topological constraints alone restrict tRNA's allowed conformational space by over an order of magnitude and strongly discriminate against formation of non-native tertiary contacts, providing a sequence independent source of folding specificity. Topological constraints also give rise to long-range correlations between the relative orientation of tRNA's helices, which in turn provides a mechanism for encoding thermodynamic cooperativity between distinct tertiary interactions. These aspects of topological constraints make it such that only several tertiary interactions are needed to confine tRNA to its native global structure and specify functionally important 3D dynamics. We further show that topological constraints are conserved across tRNA's different naturally occurring secondary structures. Taken together, our results emphasize the central role of secondary-structure-encoded topological constraints in defining RNA 3D structure, dynamics and folding.

## INTRODUCTION

Many functional RNA molecules must fold into specific, highly complex 3D structures as well as undergo precise structural dynamics in order to carry out their biological functions (1–3). Understanding how such folds and dynamics are robustly encoded by RNA's limited repertoire of four nucleobases is an outstanding challenge in biophysics. Base pairing and other weaker tertiary interactions are inherently promiscuous. Indeed, many studies have shown that the RNA-free energy landscape is rough, with folding typically proceeding through multiple metastable intermediates (4). Extensive research has uncovered thermodynamic rules that link primary sequence to secondary structure, which can be used either independently or in conjunction with experimental data to determine secondary structure (5,6). However, the thermodynamic principles that govern higher order folding specificity, stability and dynamics remain poorly understood, and even state-of-the-art prediction methods are challenged by fundamental folds such as tRNA (7).

Due to the hierarchical nature of the RNA-free energy landscape, RNA 3D folding and dynamics can be largely understood as taking place from a state possessing prefolded secondary structure. This simplification is supported by many studies (including of tRNA) showing that secondary structure typically folds both first and with greater thermodynamic stability than tertiary structure (8–12). It is worth noting that this simplification is also used by many 3D structure prediction methods, since secondary structure can be readily determined experimentally and then used as a constraint (13). Given a folded native secondary structure, the energy landscape governing 3D folding can in turn be factored into three primary components: repulsive electrostatics, attractive tertiary interactions and the energetic cost due to sterics and connectivity of organizing constituent secondary structure helices in a given conformation. Many studies have shown the importance of the former two components. Long-range tertiary interactions play a critical role in stabilizing specific conformations of helices (3,14,15), and interactions with metal cations help stabilize tertiary motifs and neutralize electrostatic repulsion between electronegative phosphate groups (16,17).

**Figure 1.**
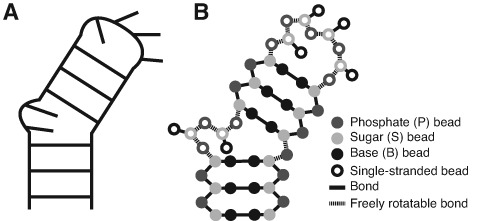
Secondary structure cartoon (**A**) and TOPRNA implementation (**B**) of a 2-nt bulge two-way junction. Filled and open circles indicate paired and single-stranded nucleotides, respectively.

More recently, studies have also begun to emphasize that the simple steric and connectivity constraints (together termed topological constraints) encoded by RNA secondary structure play an important role in defining the 3D structure and dynamics of RNA (18–22). In particular, in two-way helix-junction-helix motifs such as bulges and internal loops (e.g. Figure 1), the excluded volume of helices and connectivity constraints from the finite length of a junction's single-strands limit the orientation of helices to only 7–26% of the theoretical number of possibilities (22). Furthermore, these basic topological constraints define anisotropic free-energy landscapes that quantitatively approximate experimentally measured structural and dynamic properties of RNA bulges (18–20,22). Herschlag *et al.* hypothesized that topological constraints may also help prevent RNAs from forming non-native tertiary interactions, contributing to tertiary folding specificity (20).

Studies of topological constraints have so far focused on simple two-way junctions such as bulges and internal loops. The influence of steric and connectivity constraints on the structure and dynamics of more complex and biologically important three- and four-way junctions is less understood. Fragment assembly studies of tRNA and the adenine riboswitch indicated that interhelical linkers bias these RNAs toward native-like conformations (23). Structural surveys also identified correlations between the length of single-strands in higher order junctions and their folded conformation (24,25). However, these studies were unable to distinguish whether these observations were due to topological constraints or more complex factors such as sequence-specific base stacking. Other proposed roles for topological constraints in higher order junctions, such as their potential to contribute to RNA folding cooperativity (26), have yet to be tested. Systematic analyses of the thermodynamic contributions of topological constraints to higher order junction conformation are needed.

Studying topological constraints in higher order junctions presents unique challenges. Prior studies of topological constraints relied on *ad hoc* models that took advantage of the many simplifications afforded by two-way junctions (18–20). In bulges, the two helices are adjoined at one strand by a relatively stationary pivot. The same is true for internal loop motifs because bases tend to maximize formation of noncanonical base pairs, resulting in a bulge junction topology (18,19). This pivot-like connectivity allowed translations of the helices to be largely ignored and the finite length of the bulge linker to be modeled as a simple distance constraint. By comparison, higher order junctions lack well-defined pivots, contain multiple single-stranded loops whose behavior is difficult to model *a priori*, and have many more degrees of freedom due to the larger number of involved helices.

To address the above challenges, we recently developed the TOPological modeling of RNA (TOPRNA) coarse-grained molecular dynamics model (22). Coarse-grained molecular dynamics models have provided fundamental insights into a variety of topics in RNA folding (27,28). Similar to other models, TOPRNA uses three pseudo-atoms, or beads, to represent the base (B), sugar (S) and phosphate (P) moieties of each RNA nucleotide (nt) (Figure 1A). However, TOPRNA differs in that it is specifically designed to isolate the effects of topological constraints on RNA structure. This is achieved by treating RNA molecules as collections of self-avoiding semirigid helices linked by freely rotatable single-stranded chains (Figure 1; Supplementary Movie S1). Secondary structure base pairs are permanently bonded together, and contiguously paired regions are parameterized to adopt A-form helical conformations. By contrast, single-stranded residues experience only repulsive nonbonded interactions and are allowed to adopt any conformation that does not violate local bond and angle constraints. Electrostatic forces are also completely ignored. Thus, TOPRNA simulations very efficiently sample the 3D configurations accessible to an RNA secondary structure unbiased by primary sequence or other RNA forces. Furthermore, the frequency at which different conformations are sampled is directly related to the free energy cost (primarily entropic) posed by topological constraints.

Here, we use TOPRNA to characterize the role of topological constraints in defining the structure and dynamics of tRNA, which has long served as a paradigm for understanding RNA tertiary folding. Our results expose new features of topological constraints that are unique to higher order junctions and indicate that these constraints are harnessed by tRNA to specify its tertiary structure, dynamics and folding cooperativity.

## MATERIALS AND METHODS

### TOPRNA coarse-grained model

TOPRNA is a coarse-grained model of RNA implemented in CHARMM (29) that is parameterized to isolate the effects of connectivity and sterics on RNA 3D structure, full details of which can be found elsewhere (22). Each nucleotide is represented using three beads, one for each of the base (B), sugar (S) and phosphate (P) moieties. Local geometry is maintained through bond and angle potentials, and an improper dihedral potential is used to maintain chirality of the S-bead chiral center. Single-stranded nucleotides and segments that serve as pivots between distinct helices lack dihedral potentials, allowing them to freely rotate (Figure 1A). Secondary structure AU, GC and GU base pairs are maintained through permanent bonds, and contiguously paired regions are parameterized to adopt A-form helical conformations using backbone dihedral potentials. Potential functions follow the standard CHARMM functional form and were parameterized using a knowledge-based approach. Base pair and helical backbone dihedral potentials are sequence dependent, but local connectivity parameters are essentially identical for different residue types. Thus, the behavior of single-stranded regions is sequence independent.

Nonbonded interactions are modeled using a standard 6–12 Lennard–Jones potential and electrostatics are ignored. Other than a special exception described below, the ϵ of all beads is set to 0.01 kcal/mol. This effectively eliminates attractive interactions while preserving repulsive steric interactions. Bead radii are set to values that roughly approximate the minimum dimension of the represented moiety (e.g. the effective radii of P beads is ∼2.7 Å). This parameterization underestimates the oblong steric profile of base moities and therefore a fourth bead (M) is placed between paired B beads to fill what would otherwise be a steric gap in the middle of the pair. Base-paired B beads also experience small attractive interactions to one another, which is accomplished through selective increases in the ϵ of the LJ potential. This attractive interaction simulates base stacking within a single helix, but also marginally favors interhelical stacking across junctions (22). We emphasize that all other beads, including the B beads of single-stranded residues, do not experience this term.

### Simulation details

Initial coordinates were derived from the crystal structure (PDB 6TNA), changing modified residues to their unmodified analogs. Dihedral potentials and base pair bonds were added based on each molecule's secondary structure as described previously (22). The cut A/D-loop and cut V-loop tRNAs were obtained by removing the bond between U8(S) and A9(P) and G46(S) and U47(P), respectively. Mutant tRNAs were constructed by shifting the 6TNA residue numbering and using CHARMM to build the subsequent ‘missing’ nucleotides (sequences are shown in Supplementary Figure S9). For the VL-1 variant, coordinates for nt 46 were deleted and the upstream residue numbers shifted down. After building, all residues except for those immediately neighboring the mutation site were harmonically restrained and the system minimized. For the VS mutants, an additional round of building and minimization was used to add the V-stem and/or G26·U44 pairs after the inserted VL nts were initialized.

Restrained simulations were started from the same initial coordinates. Tertiary pairs were enforced using NOE restraints between B beads with *r*_min_ = 5.5 Å and *r*_max_ = 7.5 Å and between the associated S beads with *r*_min_ = 11 Å and *r*_max_ = 14 Å. Base triples were enforced by placing B-B and S-S NOE restraints between the tertiary nucleotide and each of the two helical nucleotides, with *r*_min_ and *r*_max_ set to ±1 Å and ±1.5 Å of the approximate B-B and S-S distances found in the crystal structure, respectively. Force constants for all NOE restraints were set to *k*_min_ = *k*_max_ = 2.0 kcal/mol/Å^2^, with maximum force asymptotes of 2.0 kcal/mol/Å. Several force field modifications were also made when restraining the tertiary G26·A44 pair, consistent with its role in extending the AC-stem. The backbone dihedrals of G26 and A44 were given potentials 1/4 the height of those used for WC-paired residues to favor A-form structure. The B beads of both G26 and A44 were also parameterized to experience a small attractive interaction to other paired B beads, as described for canonical pairs above. Finally, an M bead was added to the B bead of G26 to fill steric gaps that would otherwise exist between G26 and A44.

Both unrestrained and restrained simulations were performed using temperature replica exchange Langevin dynamics simulations with eight exponentially spaced temperature windows from 300 to 450 K. Simulations were performed in CHARMM using a 20 fs timestep and 5 ps^−1^ friction coefficient through the aarex.pl package of the MMTSB toolset (22,30). Exchange attempts were separated by 2000 dynamics steps, with acceptance ratios varying between 35 and 45%. A total of 10^9^ and 10^8^ dynamics steps per replica were performed for the unrestrained and restrained simulations, respectively, with the first 2 × 10^6^ steps of each simulation treated as equilibration and excluded from analysis. For the unrestrained simulations, this equilibration time was sufficient for the molecule to lose all memory of the starting crystal structure configuration (i.e. completely unfold). Analysis was performed on conformations recorded every 2000 dynamics steps at 300 K. Convergence was confirmed by comparing ΔG^topo^ values computed from the first 10^8^ steps of the unrestrained WT simulation to those obtained from the full 10^9^ steps; ΔG^topo^ values varied by less than 0.5 k_B_T for long-range contacts that form with ΔG^topo^<9 k_B_T.

### Measuring interhelical Euler angles

Euler angles describing the orientation between pairs of helices and the total fraction of these angles that were sampled were computed according to previously described conventions using a bin size of 10° (22,31). The H1 helix used for each pair of helices is always listed first in the text (31). The various crystal structure conformations of tRNA were obtained by searching the RNA FRABASE (32) for all 3.5 Å resolution or better X-ray structures with strand1 = ‘(((..(((’, strand2 = ‘))).(((’, strand3 = ‘)))…..(((’ and strand4 = ‘))))))’.

The fraction of global junction conformations sampled by each simulation was computed by discretizing the measured 3×(α­*_h_*, β*_h_*, γ*_h_*) angles onto a 60° 9D grid and dividing the number of sampled grid points by 108^3^ (108 is the number of nondegenerate (α­*_h_*, β*_h_*, γ*_h_*) between two helices on a 60° grid). While the number of possible grid points is significantly larger than the length of our simulations, the 499 000 snapshots of WT tRNA only sampled ∼77 000 unique 9D angles. It is likely that on a finer grid tRNA's global conformation would be substantially more constrained than estimated here.

### Measuring mutual information

Mutual information (MI) provides a general measure of correlation derived from information theory that measures the extent to which the probability distributions of the two variables are independent of one another (33). MI ranges from 0 if the distributions are completely independent, to the value of the individual distribution's Shannon entropy if they are completely dependent. The mutual information between the Euler angles of two different helices was computed as

(1) \begin{equation*} {\rm MI}(X,Y) = \sum\limits_{(X_i ,Y_i )} {P(X_i ,Y_i )\times \log } \left( {\frac{{P(X_i ,Y_i )}}{{P(X_i )\times P(Y_i )}}} \right). \end{equation*}

*X* and *Y* are the orientations of two helices measured with respect to a common H1 helix, and *P*(*X_i_,Y_i_*), *P*(*X_i_*) and *P*(*Y_i_*) are the joint and individual probabilities of the two helices adopting the specific (α­_*h*_, β*_h_*, γ*_h_*) conformations *X_i_* and *Y_i_*. Probabilities were computed using histograms with 45° bin widths. The ratio of the number of populated bins to data points ranged between 10 and 20 for the 6D histograms used to compute joint probabilities. MI overestimation due to sample size finiteness was corrected for according to ref. (33).

### Computing tertiary and coaxial stacking contacts

Two residues were considered to be in contact if the distance between their S beads was <14 Å and the residues were ≥5 apart in sequence number. Two loops were considered to be in contact if there was at least one residue–residue contact between the loops. The A/D-loop or V-loop was considered to be in contact with the D-stem if there was at least one residue–residue contact between the loop and stem.

Coaxial stacking contacts between the D- and AC-stems, the T- and A-stems and, if applicable, the V- and AC-stems, were determined using a set of criteria similar to that developed for all-atom structures (34). Our criteria are loosened to accommodate TOPRNA's coarse representation. The criteria were as follows: (i) the cosine of the angle between the base-pair-plane-normal vectors of the helix closing base pairs is ≥0.7, where the base-pair-plane was determined as the least squares fit of all of the beads of the two-paired nucleotides. (ii) The distance between the centers of mass of the two closing base pairs is ≤9 Å for directly linked helices, or ≤14 Å for helices separated by one single-stranded nt. The centers of mass were computed using only B beads. (iii) The angles between each of the base-pair-plane-normal vectors and the vector connecting the two pairs’ centers of mass are both <60°.

The energetic cost of forming a residue–residue, loop–loop or stacking contact, *c*, was computed as

(2) \begin{equation*} \Delta G^{{\rm topo}{\rm }} (c) = - k_B T\ln \left( {\frac{{P(c)}}{{1 - P(c)}}} \right). \end{equation*}

*P*(*c*) is the probability of observing the contact in a given simulation. The cost of forming joint contacts *c*_1_*, c*_2_, …, *c_n_* was computed by substituting the joint probability *P*(*c*_1_, c_2_, …, *c_n_*) into **(2)** above. The cooperativity *C* among a group of contacts was computed as

(3) \begin{equation*} C(c_1 , \ldots ,c_n ) = \frac{{P(c_1 , \ldots ,c_n )}}{{\prod\limits_n {P(c_i )} }} \end{equation*}

### Identification and analysis of best-packed conformers

Best-packed conformers from each simulation were identified by minimizing the energy function *E = n_l_ϵ_l_ + n_s_ϵ_s_*, where *n_l_* is the total number of residue–residue contacts between loops, *n_s_* is the total number of coaxial stacks, and *ϵ_l_* and *ϵ_s_* are arbitrary scaling parameters. Unless otherwise indicated, *ϵ_l_* was set to −0.6 k_B_T and *ϵ_s_* set to −3.5 k_B_T. These values were chosen based loosely on the relative interaction energies expected to be contributed by nonspecific residue–residue contacts and interhelical stacking contacts. Choice of alternative *ϵ_l_* and *ϵ_s_* values had minimal effect on the identified conformers (Supplementary Figure S4).

The entropies of the 500 conformers with lowest *E* for each simulation were computed using an approach developed to estimate protein loop entropies (35). Conformers that are close in structure to other conformers within this subensemble will have higher entropies and thus indicate enriched regions of phase space. The entropy *S_i_* of conformer *i* is computed as

(4) \begin{equation*} S_i = k_B \ln \left[ {1 + \sum\limits_{j \ne i} {\exp \left( {\frac{{ - rmsd_{i,j}^3 }}{{(10\;\;{^{\circ}_{\rm A}})^3 }}} \right)} } \right]. \end{equation*}

The sum is done over all conformers *j* ≠ *i*, with *rmsd_i,j_* the RMSD (root-mean-square-deviation) in Å between *i* and *j* using all P beads unless otherwise noted. This functional form provides a smooth measure of the number of conformers within a ∼10 Å volume of conformer *i*. The 10 Å radius was chosen based on its status as the *P* = 0.01 cut-off for structural similarity in tRNA (13).

The overall specificity of the 500 most compact conformers of each tRNA species was quantified as the entropy-weighted fraction of compact conformers that are native-like,

(5) \begin{equation*} \left\langle N \right\rangle = \frac{{\sum\nolimits_i {N_i \exp (S_i /k_B )} }}{{\sum\nolimits_i {\exp (S_i /k_B )} }}. \end{equation*}

Here, *S_i_* is the entropy of conformer *i* and *N_i_* is 0 or 1 depending on whether *i* possesses contacts between the D and T loops and does not possess contacts that preclude a native-like 3D structure. These native-inconsistent contacts were defined as contacts between any two residues of the (A/D, AC), (A/D, ACCA), (D, AC), (D, ACCA), (D/AC, ACCA), (AC, V), (AC, T), (AC, ACCA), (V, ACCA) or (T, ACCA) loops. To maintain consistency when comparing across species, residues of the A/D, V and ACCA loop residues were excluded from the RMSD calculations used to compute conformer entropies.

## RESULTS

### Topological constraints restrict tRNA global conformation and give rise to interhelical correlations

**Figure 2.**
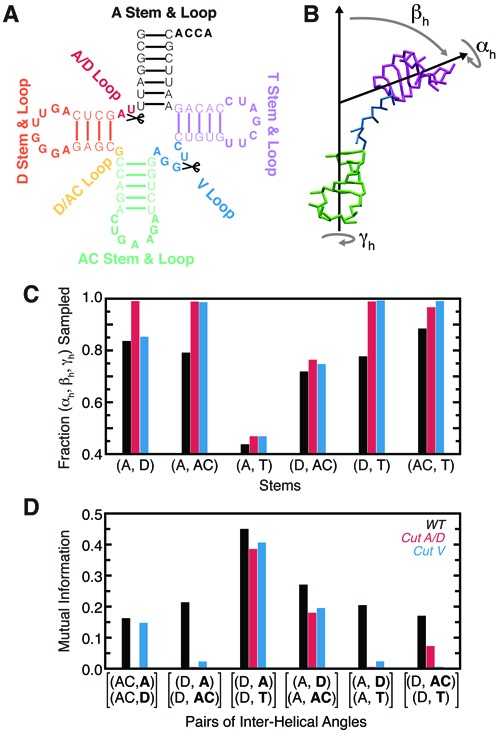
Secondary structure limits the set of global conformations accessible to WT tRNA. (**A**) Secondary structure and labeling scheme of tRNA^Phe^. Loop residues are bolded and cut locations marked. (**B**) The Euler angle convention used to describe the relative 3D orientation of RNA helices. Shown is a representative TOPRNA snapshot of the AC- and T-stems, colored as in (A), with the A- and D-stems and connecting loops not shown for clarity. (**C**) Fraction of possible (α*_h_*, β*_h_*, γ*_h_*) angles sampled between pairs of tRNA helices by the WT (black), cut A/D-loop (red) and cut V-loop (blue) simulations. (**D**) The mutual information (MI) between pairs of interhelical Euler angles measured with respect to a common reference helix. The two helices whose orientations are being correlated are bolded.

We explored the topological constraints posed by the secondary structure of tRNA^Phe^ (hereafter referred to as WT tRNA) using temperature replica exchange molecular dynamics simulations of the TOPRNA coarse-grained model (Figure 2A, Supplementary Movie S1) (22). These simulations showed good convergence after 10^8^ steps of dynamics per replica, but were extended to a total of 10^9^ steps to achieve the best sampling possible. To analyze the global conformations sampled by our simulations we use Euler angles, (α*_h_*, β*_h_*, γ*_h_*), to describe the relative orientation between pairs of tRNA helices (18,19,31). Using the AC- and T-stems as an example, Euler angles specify the twist angles α*_h_* around the T-stem and γ*_h_* around the AC-stem, while β*_h_* specifies the bend angle between them (Figure 2B). While a total of six sets of pairwise Euler angles exist between tRNA's four helices, only three sets are needed to uniquely define the orientation of three helices relative to a reference helix, arbitrarily chosen here to be the AC-stem (31).

We previously showed that in two-way junctions, topological constraints restrict the relative orientations of helices to as little as 7% of the total theoretical (α*_h_*, β*_h_*, γ*_h_*) space for 1-nt bulges and as much as 62% for an infinitely long bulge (18,19,22). The connectivity constraints posed by single-stranded bulge linkers and the pivoted topologies of these motifs also give rise to correlations between the helical twist angles α*_h_* and γ*_h_* (18,19). In WT tRNA, the four-way junction constrains the relative orientation between pairs of helices to a lesser extent (43–88%) than two-way junctions (7–62%) (Figure 2C). Interestingly, these reduced constraints allow helices to sample orientations that are forbidden in two-way junctions. For example, ∼7% of the (α*_h_*, β*_h_*, γ*_h_*) sampled between the AC- and T-stems would be inaccessible to any type of two-way junction (18,19). These unique orientations become accessible because helices in higher order junctions are no longer necessarily translationally constrained by well-defined pivots, effectively reducing their steric constraints.

As expected, the range of orientations sampled by helices increases with the length of the adjoining linker strands (Figure 2C). Thus, the A- and D-stems, which are separated by two nts, sample 82% of the (α*_h_*, β*_h_*, γ*_h_*) space, whereas the A- and T-stems, which are linked by a pivot, are limited to only 43% of their possible relative orientations. Helices that are only indirectly linked, such as the D- and T-stems, are also constrained and sample <80% of their possible relative orientations. Comparisons to simulations of tRNA with cut A/D- or V-loops (Figure 2A and C) show that helices linked by two or fewer single-stranded nts are primarily constrained by local sterics; the short linkers translationally restrain the helices such that they cannot diffuse away and are unaffected by distal cuts of the junction (Figure 2C). By contrast, the constraints on all other pairs of helices depend on junction connectivity, with ∼100% of possible conformations sampled upon junction cutting.

Although individual pairs of helices in WT tRNA are less constrained than in two-way junctions, the system as a whole is more constrained than is apparent from the pairwise analysis. As noted above, the global conformation of tRNA is described by three joint sets of Euler angles. If the helices behaved independently, the fraction of conformations sampled within this 9D angular space would equal the product of the fractions sampled by each of the three helix pairs individually. Instead, the ratio of these quantities is *r*_9D/(3×3D)_≈0.06, indicating that the orientations of tRNA's helices are coupled together. Indeed, based on mutual information (MI) measures, we observe small to moderate correlations (MI > 0) between all pairs of interhelical (α*_h_*, β*_h_*, γ*_h_*) angles (Figure 2D). These correlations are also apparent in coordinate space, with the centers of mass of different helices correlated with *R* ≈ 0.2 to *R* ≈ 0.6 (Supplementary Figure S1). Thus, once the orientation of two helices is defined, it poses constraints on the orientations of other helices either due to long-range steric effects and/or conformational restriction of the linker single-strands. For example, coaxial stacking of the D- and AC-stems anchors the termini of the A/D- and V-loops, shortening the effective linker between the A- and T-stems and thereby limiting their conformational freedom (Supplementary Figure S2). When stacked atop the AC-stem, the excluded volume of the D-stem also precludes twisted orientations of the A-stem relative to the AC-stem (Supplementary Figure S2). These correlations are significantly diminished in the cut tRNAs; the *r*_9D/(3×3D)_ ratio increases to ∼0.15 in both, and we observe dramatically reduced MI between different interhelical angles as well as reduced correlations between the centers of mass of helices (Figure 2D, Supplementary Figure S1).

Together, these correlations serve to constrain the relative orientations of WT tRNA's helices to ∼6% of the theoretically possible conformations in 9D Euler space (see methods). Thus, the ability of topological constraints to induce long-range correlations between all four helices helps compensate for the decreased constraints experienced between pairs of helices.

### Topological constraints prevent tRNA from forming non-native well-packed conformations

**Figure 3.**
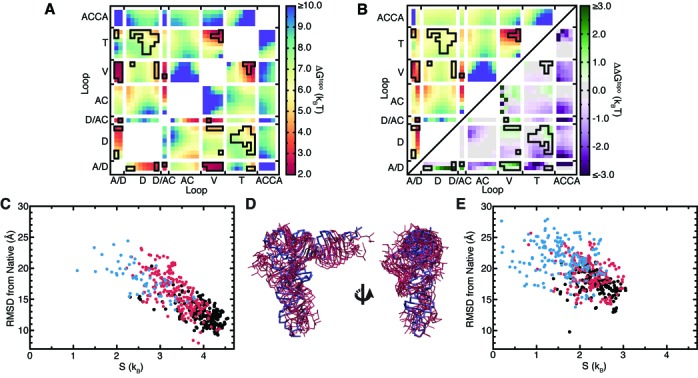
Secondary structure prevents tRNA from forming non-native tertiary contacts. (**A**) Free energy cost of forming different interloop residue–residue contacts in WT tRNA. Contacts observed in the crystal structure are outlined in black. (**B**) The free energy cost of forming different contacts upon cutting the A/D-loop. The ΔG^topo^ is shown in upper left triangle using the same color scale as (A). In the lower triangle, the ΔΔG^topo^ between the cut A/D-loop relative to WT tRNA is shown, with the color scale to the right. (**C**) Entropies and all P-bead RMSDs of the 500 best-packed conformers sampled by WT tRNA. Conformations that possess only native-consistent contacts and have D-T loop–loop contacts are colored black, those that possess only native-consistent contacts but lack D-T loop–loop contacts colored red, and those that possess native-inconsistent contacts are colored blue (see methods). Note that high entropies indicate conformers that are thermodynamically favored. (**D**) Superposition of the crystal structure (blue) and the five highest entropy best-packed conformers from the WT tRNA simulation (red). (**E**) Entropies and all P-bead RMSDs of the 500 best-packed conformers sampled by cut A/D-loop tRNA. The color scheme is the same as in (C).

Given that there are no attractive interactions in TOPRNA, the simulation of WT tRNA spends the majority of its time in entropically favored extended conformations (Movie S1). However, conformations that form long-range interloop contacts are transiently sampled, including native-like conformations (5 × 10^−4^ of conformations are <10 Å RMSD from the crystal structure). From their studies of helices linked by polyethylene glycol tethers, Herschlag *et al.* proposed that topological constraints could prevent an RNA from forming non-native tertiary contacts and thus contribute to the specificity of tertiary folding (20). To test this possibility in WT tRNA, we computed the probability *P*(*r_i_,r_j_*) at which two loop residues *r_i_* and *r_j_* come within a S-S bead distance cutoff of 14 Å, the approximate distance between two Watson–Crick (WC) paired residues. *P*(*r_i_,r_j_*) was then converted to a free energy Δ*G*^t^^opo^(*r_i_,r_j_*) that defines the energetic cost of forming the distance-dependent contact. As shown in Figure 3A, topological constraints pose a penalty as large as ∼8 k_B_T (∼5 kcal/mol at 300 K) for bringing different regions of WT tRNA into proximity. Strikingly, native tertiary contacts (outlined in black in Figure 3A) are specifically topologically favored, forming with the smallest Δ*G*^topo^ penalty (2–6 k_B_T). By contrast, non-native contacts are typically discriminated against via large Δ*G*^topo^ penalties (>7 k_B_T). It is worth noting that this several k_B_T difference is similar in magnitude to the −1 to −5 k_B_T stability of WC base pairs (36).

Comparisons to the cut tRNAs reveal that the specificity for native contacts is a unique property of WT topological constraints (Figure 3B, Supplementary Figure S3). Notably, the Δ*G*^topo^ penalty for forming non-native contacts decreases by as much as −3 k_B_T in the cut tRNAs. By contrast, the Δ*G*^topo^ of forming native ‘core’ contacts between the A/D-, V- and D-loops is increased by 1–2 k_B_T, and remains roughly unchanged for other native contacts. Thus, the ability of topological constraints to discriminate against non-native contacts is substantially reduced.

While the above results indicate that individual native tertiary contacts are specifically topologically favored in WT tRNA, they do not necessarily imply that topological constraints favor native over non-native 3D conformations. Instead, one must consider the penalty of forming many tertiary contacts ‘simultaneously.’ To explore this question, we identified the 500 WT tRNA conformers that have the maximal number of long-range contacts between single-stranded loops and inter-helical stacking interactions, which we term the ‘best-packed’ conformers (see methods). It is important to emphasize that this procedure equally weights native and non-native contacts, and ignores both sequence and local geometry. Strikingly, despite this naïve identification procedure, we find that the ensemble of best-packed conformers is highly enriched in native-like conformers (Supplementary Figure S4). The extent to which conformers are close in structure to other conformers in the best-packed subset also indicates whether they can be readily accessed, and thus are entropically favored, or conversely whether they are rarely accessed and thus entropically disfavored (35). Computing these entropies reveals that topological constraints strongly funnel WT tRNA's free energy landscape towards native-like conformations (Figure 3C). Conformers that have comparatively low RMSD from the native structure and have only native-consistent contacts (e.g. no contacts between the D- and AC-loops; see methods) have significantly higher entropies. In fact, the five conformers with highest entropy have native-state RMSDs ranging from 10.9 to 13 Å (Figure 3D). This approaches the 10 Å RMSD threshold that is a significant prediction of tRNA 3D structure (13), despite our model treating loops as freely rotatable chains and completely ignoring sequence. Together, our results strongly indicate that native secondary structure precludes formation of well-packed non-native folds, providing new insight into experimental observations that non-native RNA folds are less compact.

By contrast, applying the same procedure to the cut tRNAs shows that best-packed conformations are significantly less enriched in native-like conformations and are less funneled toward the native state (Figure 3E, Supplementary Figure S5). In combination with Figure 3C and Supplementary Figure S3, these findings lead to the prediction that cutting one strand in tRNA should decrease thermodynamic stability. Significantly, this agrees with experiments showing that cuts anywhere within the A/D-loop or at the V-loop termini catastrophically disrupt the ability of tRNA to fold (37). The same experiments also showed that cuts to D/AC-loop and T/A-linker also reduced folding to a lesser degree; while not simulated here, this is consistent with our expectation that such cuts should similarly disrupt WT tRNA's topological constraints. However, in what is likely a result of stabilization afforded by tertiary base-triples, cuts to the interior of the V-loop did not significantly affect folding even though we predict they should comparably disrupt topological constraints (37). As a whole, these observations support that topological constraints constitute an important component of the tRNA folding landscape, emphasizing that tertiary interactions and electrostatics also play key roles. Notably, the particular severity of A/D-loop cuts is likely due to the >3 k_B_T increase in the Δ*G*^topo^ of forming the crucial U8·A14 tertiary contact in addition to the overall decrease in topological constraints (Figure 3B).

### Topological constraints render some tertiary interactions redundant and help direct tRNA dynamics along specific pathways

**Figure 4.**
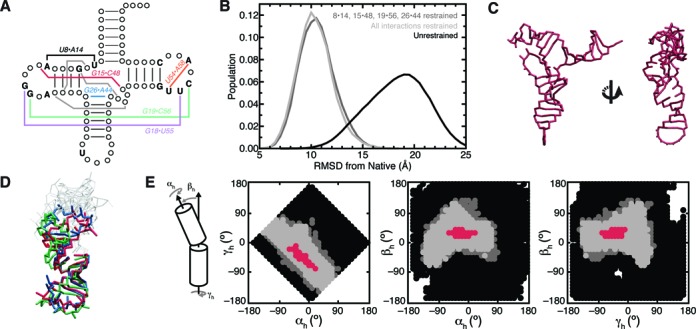
Tertiary interactions confine WT tRNA to native-like conformations. (**A**) Diagram of conserved tRNA residues and tertiary interactions. Residues conserved in <90% of tRNA species are indicated by circles (38). Conserved tertiary interactions are labeled and semiconserved base triples are drawn as gray lines. (**B**) RMSD distributions of simulations of unrestrained WT tRNA (black), WT tRNA restrained by all nine conserved tertiary interactions (tRNA_9R_; light gray) and WT tRNA restrained by the four non-redundant interactions (tRNA_4R_; dark gray). (**C**) The average structure of tRNA_9R_. (**D**) Three representative structures from the tRNA_9R_ simulation illustrating the orientations sampled between the D- and AC-stems. Structures are superimposed by the AC stem. Residues of the A- and T-stems and connecting loops are not colored for clarity. (**E**) 2D projections of the (α*_h_*, β*_h_*, γ*_h_*) angles sampled between the AC- and D-stems. Angles only sampled by unrestrained tRNA are shown in black; angles sampled by both unrestrained and tRNA_4R_ are shown in dark gray; and angles sampled by tRNA_9R_, tRNA_4R_ and unrestrained WT tRNA are shown in light gray. Red points correspond to angles measured from 109 different tRNA crystal structures. A reference cartoon of the three angles is shown at left. Note that as discussed in the text, examining only one pairwise set of (α*_h_*, β*_h_*, γ*_h_*) angles provides an incomplete picture of the extent to which topological constraints confine tRNA conformation.

We next examined the consequences of combining the specific conserved tertiary interactions of tRNA with topological constraints (Figure 4A). Still treating loop residues as freely rotatable chains, we performed a TOPRNA simulation of WT tRNA restrained by these nine tertiary interactions using simple residue–residue distance restraints (tRNA_9R_). tRNA_9R_ is effectively constrained to only native-like global conformations (Figure 4B); the average structure is ∼8 Å RMSD from the crystal structure (Figure 4C), and conformations with high native RMSDs can be attributed to global twisting and bending motions of helices around this average structure (Supplementary Figure S6). Compared to the distribution of best-packed conformations in the unrestrained simulation of WT tRNA (Supplementary Figure S4), non-native conformations are eliminated and the population of low RMSD conformations is increased in tRNA_9R_. Thus, native tertiary interactions effectively stabilize the set of best-packed conformations favored by topological constraints and further funnel them toward the native conformation. The need for only native secondary structure and several tertiary interactions to define macroscopic structure in this manner may explain why there are comparatively few sequence constraints on other regions of tRNA (Figure 4A) (38,39).

We also examined whether the full set of tertiary interactions is needed to restrict WT tRNA to native conformations. Additional restrained simulations indicated that the base triple, U54·A58 and either the G18·U55 or G19·C56 restraints could all be removed without an increase in the mean native RMSD (tRNA_4R_; Figure 4B). Of the remaining ‘nonredundant’ U8·A14, G15·C48, G26·A44 and G19·C56 restraints, all but the U8·A14 restraint could be singly removed with the mean native RMSD increasing by only 1–2 Å (not shown).

These results indicate that, at the coarse level of our models, topological constraints render some of tRNA's tertiary interactions structurally redundant. While the favorable interaction energies contributed by the ‘nonredundant’ contacts surely play an important role in stabilizing the native fold, they are unnecessary for defining tRNA global architecture. A caveat is that the above simulations were begun from the native conformation, and thus the full set of interactions could still be necessary to specify the native state from unfolded conformations. We tested this by hierarchically ‘folding’ tRNA molecules from random initial configurations using only the four nonredundant tertiary restraints (see Supplementary Information). Repeating this 200 times and clustering the product ‘folds’ revealed that the most populous cluster was native (Supplementary Figure S7). Thus, coupled with the entropic bias of topological constraints, the nonredundant interactions are sufficient to specify global native structure. Notably, the apparent redundancy in tRNA's tertiary interaction network is consistent with evidence that not all conserved tertiary pairs are required for folding. Some cytosolic and many mitochondrial tRNAs lack a subset of conserved pairs (39,40), and individual ablations (39,41–43) or complete reengineering (44) of the tertiary interactions of canonical tRNAs do not inhibit function. Our results also complement prior studies that showed that tRNA 3D structure can be predicted based on these nonredundant interactions (45–49).

As noted above, a marked feature of both the tRNA_4R_ and tRNA_9R_ simulations is that tRNA retains significant structural flexibility. This flexibility would likely be reduced upon inclusion of energy terms beyond topological constraints. Nevertheless, it is interesting to note that this flexibility appears to be directed along specific motional modes that are qualitatively similar to those identified by more rigorous studies of tRNA dynamics (50–53). These include global bending and twisting motions of the two arms of the tRNA ‘L,’ and bending and twisting of the D- and AC-stems relative to one another (Figure 4D and E; Supplementary Figures S6 and S8). It is well known that such motions are integral to tRNA function, including recognition by aminoacyl synthetases and initial selection and translocation on the ribosome (53,54). Thus, similar to how topological constraints and a few tertiary interaction constraints are sufficient to encode macroscopic structure, these constraints are also sufficient to macroscopically define biologically important dynamics.

### Topological constraints give rise to folding cooperativity

The apparent correlations between the orientation of WT tRNA helices (Figure 2D, Supplementary Figure S1) together with the redundancy in tRNA's tertiary interaction network (Figure 4B) suggests that topological constraints may also provide a basis for folding cooperativity. Here, formation of a subset of native tertiary interactions imposes greater structural confinement and thus reduces the entropic penalty for forming remaining native tertiary interactions. This mechanism could help explain the tertiary folding cooperativity exhibited by tRNA (55) and other RNAs (26,56), and parallels the explanation given for folding cooperativity in proteins (57).

**Figure 5.**
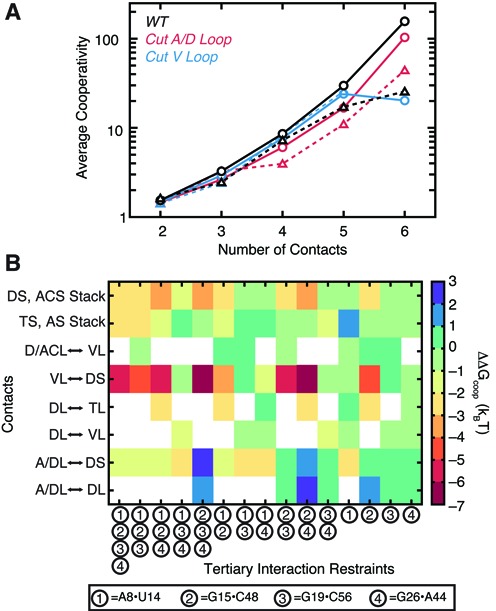
tRNA's tertiary interaction network is cooperative. (**A**) The mean cooperativity of jointly forming *n* number of loop–loop or stacking contacts, averaged over all combinations of native contacts (solid line, circles) or combinations containing at least one non-native contact (dashed line, triangles). Results are shown for the WT (black), cut A/D-loop (red) and cut V-loop (blue) simulations. Combinations that were observed ≤10 times were excluded from the averages. (**B**) The thermodynamic cooperativity among tRNA's tertiary interactions, computed with Equation (6). Restrained tertiary interactions are numbered on the x-axis according to the shown key. Loop–loop, loop–stem and stacking contacts for which cooperativities were measured are shown along the y-axis. Cooperativities are not computed for loops that have an active restraint placed between them.

We quantified this cooperativity from our unrestrained simulations by computing the ratio of the joint probability *P*([*l_i_, l_j_*], [*l_k_,l_m_*], *s_i_*, …) of forming contacts between different pairs of loops [*l_i_, l_j_*] and [*l_k_,l_m_*] and interhelical stacks *s_i_*, to the product of the individual probabilities (see methods). This coarse analysis confirms that, on average, different contacts form cooperatively, and that cooperativity increases as more contacts are formed (Figure 5A). Notably, combinations of six native contacts form on average with a ∼200× greater probability than if they were independent. The cooperativity among native contacts is also as much as 6× higher than between combinations of non-native contacts. Thus, topological constraints also help prevent non-native contacts from forming cooperatively. By contrast, the cut tRNAs exhibit a modest decrease in cooperativity, and perhaps more importantly, a narrower difference in the cooperativities of native versus non-native contacts (Figure 5A). The only modest decrease indicates that much of the observed cooperativity is a natural function of the arrangement of secondary structure elements in the native fold versus connectivity constraints posed by the A/D and V-loops.

We also computed the energetic consequences of cooperativity on native folding in WT tRNA by taking the difference between the Δ*G*^topo^ of forming a set of contacts {TC} in a simulation restrained by tertiary interactions {TI}, relative to the Δ*G*^topo^ of forming {TC} without restraints:

(6) \begin{equation*} \begin{array}{*{20}l} {\Delta \Delta G_{{\rm coop}} (\{ {\rm TC}\} ,\{ {\rm TI}\} )} \\ { = \Delta G^{{\rm topo}} (\{ {\rm TC}\} )_{\{ {\rm TI}\} {\rm restrained}} - \Delta G^{{\rm topo}} (\{ {\rm TC}\} )_{{\rm unrestrained}} .} \\ \end{array} \end{equation*}

In agreement with our analysis of the unrestrained simulations, single interactions by themselves only weakly influence the stability of other contacts (Figure 5B). However, as more interactions are restrained, formation of other contacts becomes increasingly energetically favored. Particularly notable is that the G15·C48 tertiary pair contributes up to −7 k_B_T to the stability of contacts between the V-loop and the D-stem, potentially explaining why the base triples that form between these regions have few sequence constraints (58), and consistent with the above observed redundancy of these triples. The G15·C48 pair also promotes interhelical stacking, both individually (−3 k_B_T <ΔΔ*G*_coop_<0 k_B_T) and more strongly in conjunction with other tertiary pairs (−4 k_B_T <ΔΔ*G*_coop_<−2 k_B_T). By contrast, the U8·A14 pair generally disfavors formation of additional contacts, and vice versa, other tertiary restraints disfavor formation of A/D- to D-loop contacts. This anticooperativity arises because of the large increase in steric constraints associated with bringing the A- and D-stems into close proximity; tRNA conformations that have both the U8·A14 pair and other native contacts are thus entropically disfavored. The inability of U8·A14 to be stabilized by other interactions, coupled with its importance for confining tRNA to its native-state (see above), may explain its particularly strong evolutionary conservation (38,39).

### Topological constraints are conversed across diverse tRNA secondary structures

**Figure 6.**
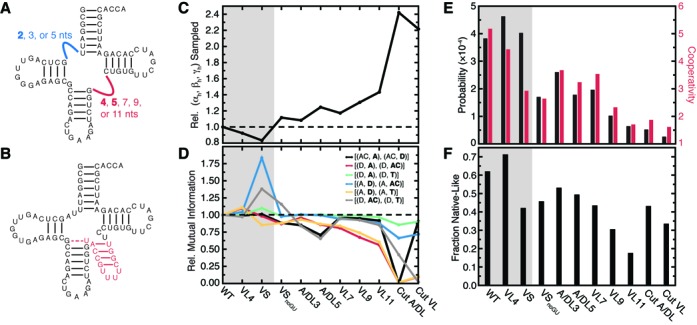
Naturally occurring tRNA secondary structures conserve topological constraints. (**A**) Class I tRNAs. The A/D and V-loop are shown as blue and red lines, respectively, with tested length variations labeled. Naturally observed lengths are bolded. Full sequences are shown in Supplementary Figure S9. (**B**) Example Class II tRNA. Inserted V-stem is shown in red, and G26·U44 pair shown by a dashed line. Note the additional D-loop nt (gray) and 3-bp D-stem. (**C**) Relative fraction of 3×(α*_h_*, β*_h_*, γ*_h_*) interhelical conformations sampled by different tRNAs compared to WT. (**D**) Mutual information between different pairs of interhelical Euler angles relative to WT tRNA. The helices whose orientations are being correlated are bolded in the key. (**E**) The probability and cooperativity of jointly forming loop–loop contacts between the D and T loops and both native interhelical stacks. (**F**) The fraction of 500 best-packed conformers that possess native-like folds, weighted by entropy as described in methods. The gray background in (C–F) is used to highlight natural tRNA variants.

While the large majority of tRNA species share the classic cloverleaf secondary structure explored above, there are several commonly observed variations (39). The most common variation involves a decrease in the length of the V-loop from 5-nt to 4-nt (Figure 6A). Not too surprisingly, TOPRNA simulations of a tRNA with 4-nt V-loop (VL4) reveals that this molecule is similarly if not even more confined by topological constraints, and has similar native-state specificity (Figure 6).

A more dramatic variation is found in so-called Class II tRNAs, where the V-loop is replaced by a several base pair long V-stem (Figure 6B). This is normally accompanied by replacement of the tertiary G26·A44 pair atop the AC-stem with a more stable G26·U44 pair, as well as several changes to the D-loop (Figure 6B) (38). Despite these secondary structure differences, Class II tRNAs fold to a common 3D structure through poorly understood mechanisms. Strikingly, TOPRNA simulations of a Class II tRNA (VS) reveal that topological constraints are conserved, with the overall number of interhelical conformations sampled decreasing by ∼17% and the probability of jointly forming stacking interactions and D- and T-loop contacts increasing by ∼5% (Figure 6C and E). However, native contacts form less cooperatively and the best-packed conformations of VS are substantially less native-specific, suggesting that the folding landscape of this tRNA may be more complex than Class I species (Figure 6).

Inversely, we find that changes in tRNA secondary structure that would be expected to disrupt topological constraints are evolutionarily disfavored. While an entire stem can replace the V-loop in Class II tRNAs, it is very rare to observe tRNAs with single-stranded V-loops longer than 5-nts (Figure 6A) (38,39). Consistent with the negative selection of such secondary structures, simulations of Class I tRNAs with 7-nt (VL7), 9-nt (VL9) and 11-nt (VL11) long V-loops indicate that these changes decrease topological constraints (decrease by 17–43%; Figure 6C) and reduce native-state specificity (decrease by 33–75%; Figure 6E, F). By comparison, Class II tRNAs preserve WT-like topological constraints by sequestering additional V-loop nucleotides into a hairpin.

Topological constraints also help explain why the noncanonical G26·A44 pair of Class I tRNAs is replaced in most Class II tRNAs by a more stable G26·U44 pair (Figure 6B) (38). Removing the G26·U44 base-pair from VS (VS_noGU_) reduces topological constraints by ∼30% compared to VS and significantly reduces the probability of forming native tertiary contacts (Figure 6). This result also helps explains why U44 is often 2′-O-methylated in Class II tRNAs that contain G26·U44 pairs. Significantly, although this modification is thought to function by locally stabilizing the G26·U44 pair, its absence has been shown to globally destabilize Class II tRNAs (59,60).

Finally, we explored the effects of lengthening the A/D-loop, which is universally conserved in all tRNAs to be ≤2 nt long (Figure 6A) (38,39). Simulations of Class I tRNAs with 3-nt (A/DL3) and 5-nt (A/DL5) A/D-loops reveal that topological constraints and native specificity are modestly decreased in these molecules (8–50%, depending on tRNA and property; Figure 6). While consistent with the evolutionary preference for 2-nt or less A/D-loops, these modest decreases are likely insufficient to explain why such secondary structures are never observed in nature. Among many possible explanations, additional nucleotides may disrupt the native tertiary interactions that form between the A/D-loop and the D-loop and stem, or lead to misfolded secondary structures.

## DISCUSSION

Our results show that topological constraints encoded at the secondary structure level provide a robust strategy for encoding macroscopic properties of RNA 3D structure and dynamics. In tRNA, topological constraints serve as a source of negative design (61,62) by imposing significant penalties on the formation of non-native tertiary contacts. This is in strong agreement with the hypothesis of Herschlag *et al.* that topological constraints provide a mechanism for circumventing the limited specificity of RNA's nucleotide alphabet (20). By coupling the orientation of helices together over long length scales, topological constraints also allow a tertiary interaction formed in one region of an RNA to influence the likelihood of forming additional distant tertiary interactions. This provides a source of folding cooperativity that helps stabilize tRNA's tertiary structure. It is important to emphasize that topological constraints are largely sequence independent. Thus, for tRNA, these properties are inherited by any species that maintains an appropriate secondary structure. This may help free the primary sequence of tRNA to vary according to other functions orthogonal to folding (54).

As discussed above, our findings are consistent with, and can help explain, many prior experiments on tRNA. It is particularly satisfying that our results help explain why evolution has conserved the Class I and Class II isoforms of tRNA secondary structure, but strongly selects against secondary structures with V-loops containing more than five single-stranded residues. Our results may also explain the thermodynamic coupling between secondary and tertiary structure folding that is observed in some tRNAs (10,63–67). Experimental (11) and computational (12) studies of tRNA suggests that folding of native secondary structure nucleates tertiary structure folding. While not true for all RNAs (68,69), this is consistent with our hypothesis that secondary structure exerts a powerful influence on tertiary structure stability. This is further supported by experiments showing that stabilizing secondary structure can rescue folding of tRNA mutants with disrupted tertiary interactions (67), and by the clear implication that increased GC sequence content stabilizes thermophilic tRNA species (39,70).

A growing body of literature has suggested the importance of junction secondary structure to the folding of other RNAs. Correlations between the length of single-strands in junctions and their folded conformation have been identified and used with some success to predict RNA 3D conformation (24,25,71–74). Paralleling our results, Sim and Levitt found that fragment assembly models built from secondary structure were biased toward the native conformation (23). Experiments have also shown that the junction of the hairpin ribozyme modulates the thermodynamics of tertiary folding, primarily by altering the entropic cost of folding (75–78). However, the physical basis for these observations has remained unclear. Combined with prior studies of two-way junctions (18–20,22), our work indicates that topological constraints provide a free energy based framework for understanding the link between secondary structure and 3D conformation.

The ability of topological constraints to discriminate against non-native tertiary interactions and encode cooperativity may be particularly important for large RNAs. Notably, cooperativity similar to what we find in tRNA has been shown to be critical to the tertiary structure stability of large RNAs (26,56,79). These RNAs also often utilize multiple identical tertiary interaction motifs to stabilize their 3D folds, implying that factors beyond sequence code for their specificity (80,81). Large RNAs also fold through native-like compact intermediates that lack fully formed tertiary interactions (82), and are stabilized by molecular crowders that nonspecifically favor compact 3D conformations (83,84). In a unique case, a segment of the HIV-1 genome RNA was shown to adopt a well-defined solution structure despite not having well-defined tertiary contacts (85). These observations are consistent with secondary structure providing an inherent source of 3D folding specificity. Although not explored here, we note that topological constraints could also play important kinetic roles in RNA folding. For example, progressive formation of helices and accompanying topological constraints could bias folding along specific pathways (21,86).

Clearly, topological constraints operate only on a coarse level. Other forces, including electrostatics and sequence-specific attractive interactions, must be considered in order to achieve an atomistic level of understanding of RNA structure and dynamics. The assumption of fixed RNA secondary structure used in our study also makes it difficult to explore thermodynamic coupling between secondary structure and tertiary structure folding. However, the additivity of free energy ensures that the specificity and cooperativity encoded by topological constraints will translate to real RNAs. For promiscuous tertiary interactions, the differential energetic costs posed by topological constraints could be a primary factor in determining the folding outcome. Furthermore, the framework afforded by topological constraints offers many useful applications. This includes indentifying conserved 3D structural and dynamic elements hidden in what may otherwise be very different RNA secondary structures, such as shown here for Class I and Class II tRNAs. Considering topological constraints may also aid the rational design of RNAs with novel properties and help improve structure prediction algorithms (87). We anticipate that studies exploring these potential applications will help achieve a deeper understanding of how RNAs fold and carry out their biological functions.

## SUPPLEMENTARY DATA

Supplementary Data are available at NAR Online.

SUPPLEMENTARY DATA
