# Supplementary material for: Topological constraints are major determinants of tRNA tertiary structure and dynamics and provide basis for tertiary folding cooperativity
Source: Nucleic Acids Res. 2014 Sep 12;42(18):11792–804. doi: 10.1093/nar/gku807 (PMC4191394; doi:10.1093/nar/gku807)
Supplement: SUPPLEMENTARY DATA [file supp_42_18_11792__index.html]

Topological constraints are major determinants of tRNA tertiary structure and dynamics and provide basis for tertiary folding cooperativity — SUPPLEMENTARY DATA 

# Topological constraints are major determinants of tRNA tertiary structure and dynamics and provide basis for tertiary folding cooperativity

## SUPPLEMENTARY DATA

**Files in this Data Supplement:**

- SUPPLEMENTARY DATA
- SUPPLEMENTARY DATA
