# Supplementary material for: Topological constraints are major determinants of tRNA tertiary structure and dynamics and provide basis for tertiary folding cooperativity
Source: Nucleic Acids Res. 2014 Sep 12;42(18):11792–804. doi: 10.1093/nar/gku807 (PMC4191394; doi:10.1093/nar/gku807)
Supplement: SUPPLEMENTARY DATA [file supp_gku807_nar-01616-r-2014-File009.pdf]

SUPPORTING INFORMATION

Topological Constraints are Major Determinants of tRNA Tertiary Structure and Dynamics and Provide Basis for Tertiary Folding Cooperativity

Anthony M. Mustoe[a], Charles L. Brooks III[a,b,*], and Hashim M. Al-Hashimi[c,*]

Departments of Biophysics[a] and Chemistry[b], University of Michigan, Ann Arbor, MI 48109-1055, USA

[c]Department of Biochemistry, Duke University Medical Center, Durham, NC 27710

[*]Corresponding authors: Email: hashim.al.hashimi@duke.edu and brookscl@umich.edu

SUPPLEMENTARY METHODS AND ANALYSIS

Measuring correlations between the centers of mass of different helices

To correlate the centers of mass (COM) of two helices, H_i and H_j, a reference helix H_r ($H_r \neq H_i \neq H_j$) was chosen as an internal reference frame. Global translations and rotations of the molecule were removed by superimposing the three junction-closing base pairs of H_r. H_i and H_j COM time series were then computed using the S and P pseudo-atoms of the three junction-closing base pairs of each helix, and the canonical correlations of these time series were computed using the CCA library of R. The mean of the three orthogonal canonical correlation coefficients was used as the correlation expected along an arbitrary direction in 3D space.(1) This was repeated for both possible choices of H_r, as different H_r's remove global rotations and translations of the molecule with varied efficacy. The minimum of the two obtained mean correlations was chosen as the best representative. Time-randomizing the two COM variables and repeating the correlation analysis confirmed significance; in all cases, these time-randomized COM variables had $R \leq 0.005$.

Folding tRNA with four restraints from an initially unfolded conformation

The potential for G26•A44, U8•A14, G15•C48, and G19•C56 tertiary restraints to determine tRNA's native 3D structure from unfolded conformations was probed through simulations that hierarchically 'folded' the molecule from random configurations of the secondary structure. The restraints were enforced hierarchically in the order listed above with r_{min} and r_{max} identical to the values used for other restrained simulations. Force constants were initially set to kmax=kmin=0.1 kcal/mol/Å^2 and fmax=0.5 kcal/mol/Å, with the system allowed to equilibrate over 2×10^6 steps of Langevin dynamics at 300 K after the addition of each restraint. After all restraints were added, the system was allowed to further equilibrate over 10^5 steps of dynamics performed at each of 500 K, 450 K, 400 K, 350 K, and 300 K. Restraint force constants were then increased to kmax=kmin=4.0 kcal/mol/Å^2 and fmax=4.0 kcal/mol/Å and the system allowed to equilibrate over an additional 10^5 steps of dynamics at 300 K. Production simulations were

performed at 300 K for a total of 10^6 dynamics steps, recording conformations every 10^3 steps, and the simulation-average structure computed.

In total, this procedure was performed for 200 different initial configurations, yielding 200 average structures. The generated average structures were then clustered according to the similarity of their measured $3\times(\alpha_h, \beta_h, \gamma_h)$ angles using the fixed-radius ART-2' clustering algorithm,(2) modified to use cluster medoids instead of cluster means. Distances between two conformations *i* and *j* were computed as

$$d_{ij} = \tfrac{1}{3}\sum_{n=1}^{3}\left(\theta_{ij}\right)_n, \qquad \textbf{[S2]}$$

where $(\theta_{ij})_n$ is the single-axis rotation amplitude(3) needed to bring the *n-th* $(\alpha_h, \beta_h, \gamma_h)$ of conformation *i* into concordance with the *n-th* $(\alpha_h, \beta_h, \gamma_h)$ of conformation *j*, and the clustering radius was set to 60°. This radius was chosen due to the observation that two different pairs of coaxially stacked helices, sourced from our previously constructed database of coaxially stacked helices within the PDB,(4) may possess $(\alpha_h, \beta_h, \gamma_h)$ differing by as much as θ_{ij}=60°. Thus, a choice of 60° should allow two such equivalent conformations to cluster together.

Multiple independent clusterings computed from different initial orderings of the structures consistently yielded 7 clusters, with the most populous cluster (35%-41% of the population) always comprised of native conformations. The next most populous cluster contained no more than ~26% of the average structures. The members of non-native clusters represent different topoisomers that arise from the non-equilibrium folding methodology. While able to fulfill the four applied distance restraints, these structures were clearly not feasible RNA folds. For example, only structures in the native cluster were able to form any type of inter-helical stacking interactions.

SUPPLEMENTARY REFERENCES

1. Briki, F. and Genest, D. (1994) Canonical analysis of correlated atomic motions in DNA from molecular dynamics simulation. *Biophys. Chem.*, **52**, 35-43.

2. Karpen, M.E., Tobias, D.J. and Brooks, C.L., III. (1993) Statistical clustering techniques for the analysis of long molecular dynamics trajectories: analysis of 2.2-ns trajectories of YPGDV. *Biochemistry*, **32**, 412-420.
3. Bailor, M.H., Mustoe, A.M., Brooks, C.L., III and Al-Hashimi, H.M. (2011) 3D maps of RNA interhelical junctions. *Nat. Protoc.*, **6**, 1536-1545.
4. Mustoe, A.M., Bailor, M.H., Teixeira, R.M., Brooks, C.L., III and Al-Hashimi, H.M. (2012) New insights into the fundamental role of topological constraints as a determinant of two-way junction conformation. *Nucleic Acids Res.*, **40**, 892-904.

SUPPLEMENTARY FIGURES

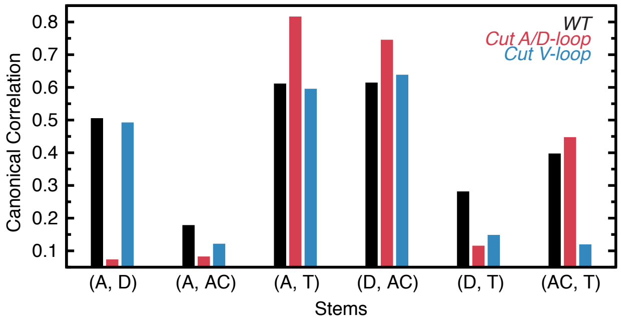

Figure S1: Canonical correlations measured between the centers of mass of different tRNA helices. Correlations were measured as described in supplementary methods. The increased correlations between the (A, T), (D, AC), and (AC, T) stems of the cut tRNAs arise because global translations and rotations of these molecules were less effectively removed by reference helix superpositions. These residual global motions inflate the canonical correlation measured between helices. Note that these increased correlations are not observed by mutual information measures (main text Figure 2D).

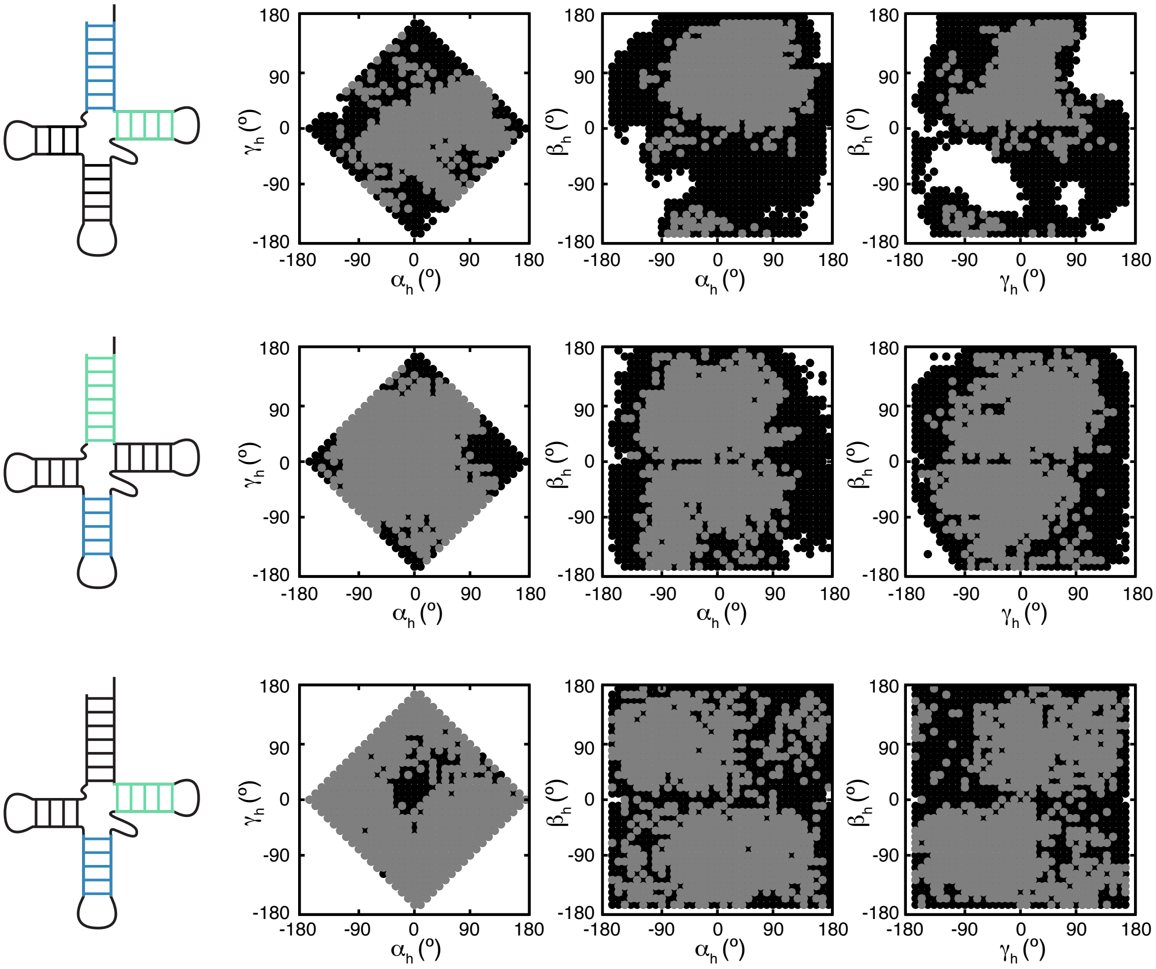

Figure S2: 2D projections of the (α_h, β_h, γ_h) angles sampled between stems of WT tRNA when the D- and AC-stems are coaxially stacked (gray points), superimposed on top of all (α_h, β_h, γ_h) angles sampled by unrestrained WT tRNA (black points). Thus, black points denote otherwise accessible regions of (α_h, β_h, γ_h) space that are inaccessible when the D- and AC-stems are stacked. Cartoons on the left indicate the plotted inter-helical angle, with stems highlighted in blue denoting the H1 reference helix and stems highlighted in green denoting the H2 helix.(3) Cutoffs of $|\beta_h|<20$ and $|\alpha_h + \gamma_h|<50$ between the D- and AC-stems were used to identify coaxially stacked conformations.

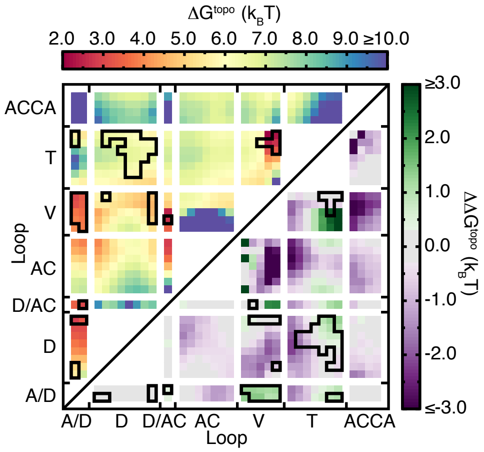

Figure S3: The free energy cost of forming different contacts upon cutting the V-loop. The ΔG^{topo} is shown in upper triangle. The $\Delta\Delta G^{topo}$ between the cut V-loop relative to WT tRNA is shown in the lower triangle.

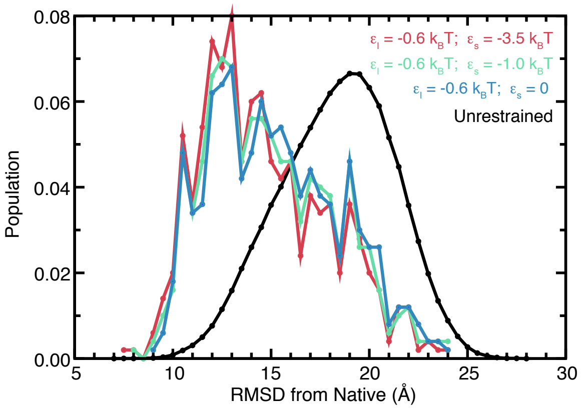

Figure S4: Histogram of the all-P RMSD from the 6TNA crystal structure of all conformers sampled by the unrestrained simulation of WT tRNA (black), and of the 500 best-packed conformers identified using different values of ε_l and ε_s (colored; see key).

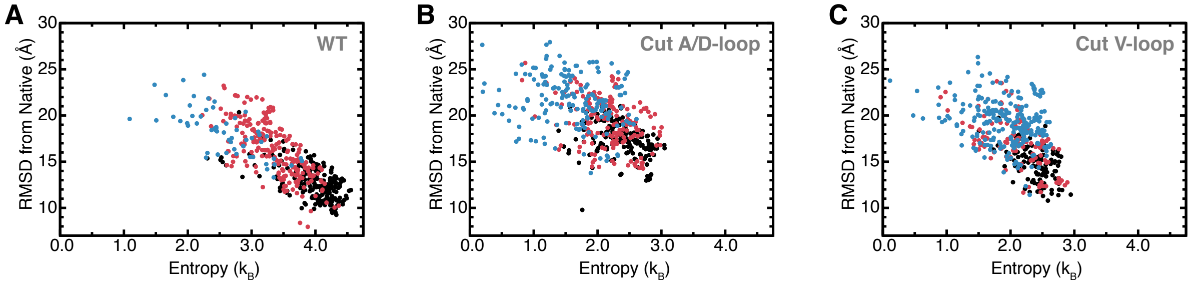

Figure S5: Entropies and RMSDs of the 500 best-packed conformers at ε_l=-0.6 k_BT and ε_s=-3.5 k_BT for (**A**) WT, (**B**) cut A/D-loop, and (**C**) cut V-loop tRNAs. Conformations that possess only native-consistent contacts and have contacts between the D- and T-loops are shown in black, conformations that possess only native-consistent contacts but do not have D-T loop contacts are shown in red, and conformations possessing native-inconsistent interactions are shown in blue (see main text methods). Note that **A** and **B** are identical to main text Figures 2C and 2E.

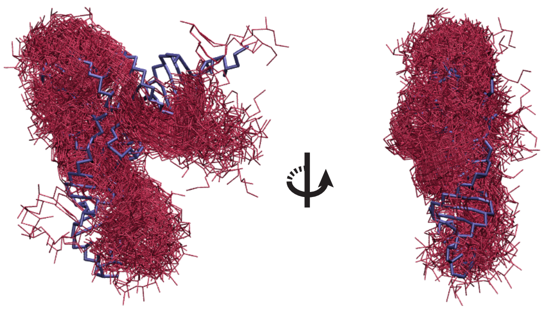

Figure S6: Conformations with >15 Å RMSD from the native structure sampled by the simulation of WT tRNA restrained by all nine tertiary interaction restraints (red). Shown are 50 randomly selected conformations from 1249 total with >15 Å RMSD. These structures are superimposed on the 6TNA crystal structure (blue).

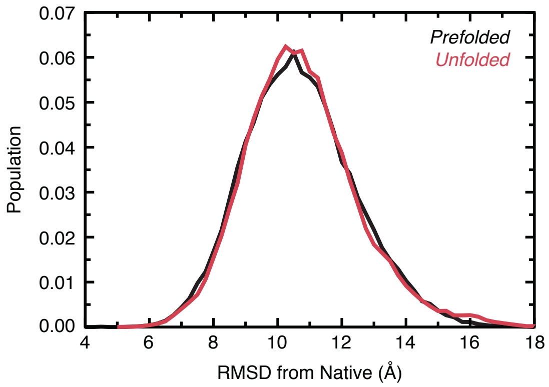

Figure S7: RMSD distributions of restrained simulations of WT tRNA started from 'prefolded' initial coordinates and from 'unfolded' initial coordinates that were folded using a hierarchical folding scheme (see supplementary methods). The 'unfolded' curve represents the aggregate RMSD distribution of the production runs of members of the largest cluster. G26•A44, U8•A14, G15•C48, and G19•C56 were restrained in both simulations.

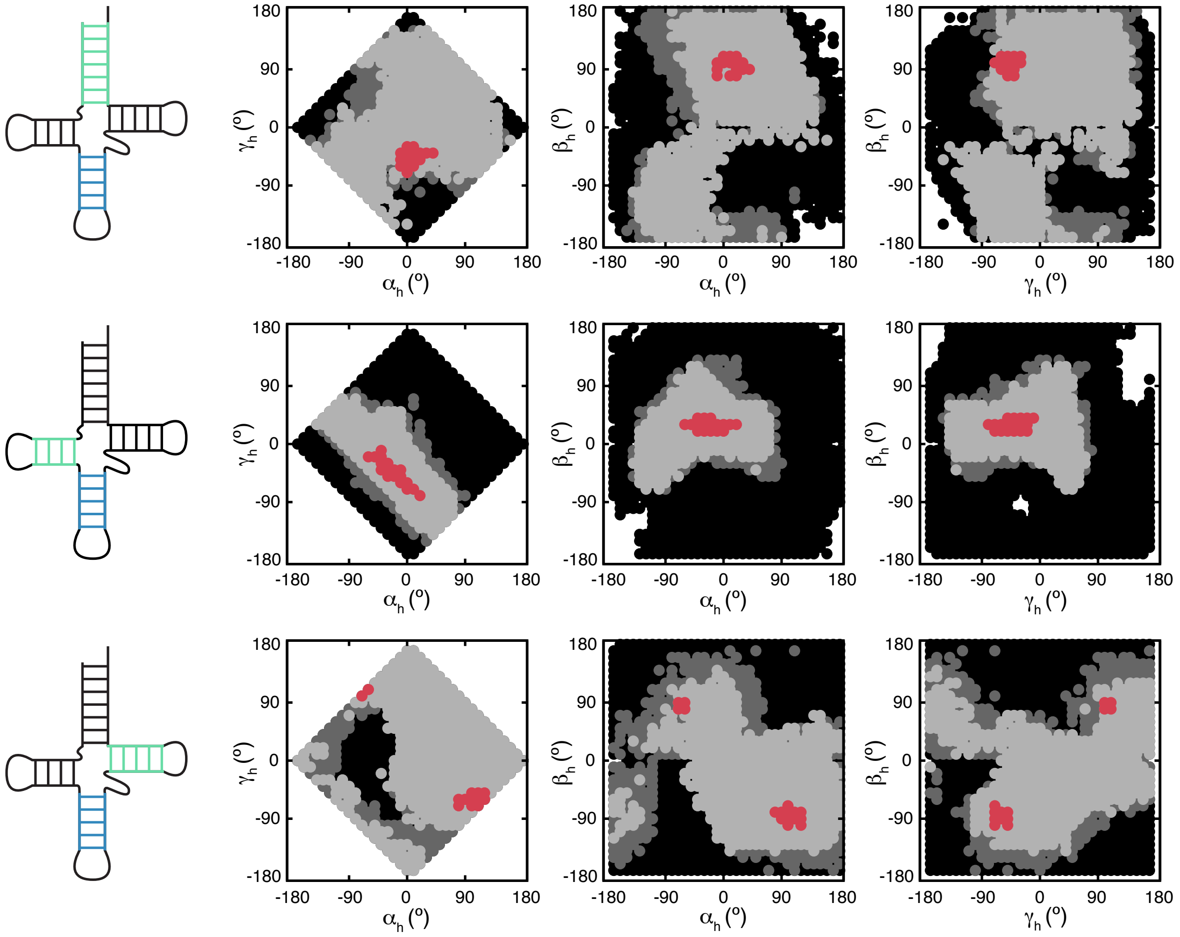

Figure S8: 2D projections of the (α_h, β_h, γ_h) angles sampled between different pairs of helices are shown for simulations of WT tRNA that are unrestrained, restrained by the non-redundant interactions G26•A44, U8•A14, G15•C48, and G19•C56, and fully restrained by all tertiary interactions shown in main text Figure 3A. Angles only sampled by unrestrained tRNA are shown in black, angles sampled by both unrestrained and non-redundantly restrained tRNA are shown in dark gray, and angles sampled by all three simulations are shown in light gray. Red points correspond to angles measured from 109 different tRNA crystal structures. Cartoons on the left indicate the plotted inter-helical angle, with stems highlighted in blue denoting the H1 reference helix and stems highlighted in green the H2 helix.(3)

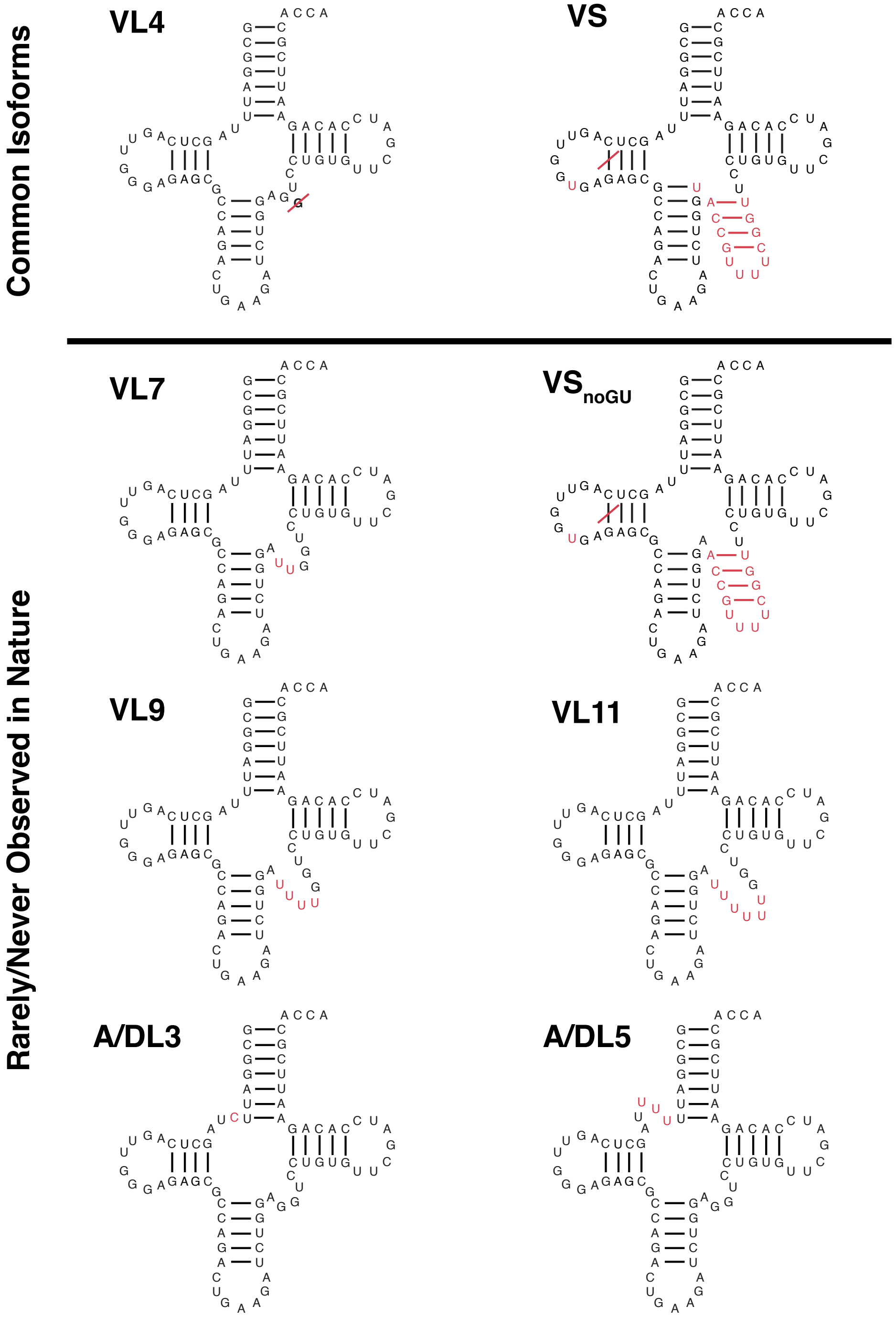

Figure S9: Detailed sequences and secondary structures of the different simulated tRNA variants. Inserted features compared to WT tRNA are shown in red, and deleted features are marked by a red strike.
